# A suite of ^19^F based relaxation dispersion experiments to assess biomolecular motions

**DOI:** 10.1007/s10858-020-00348-4

**Published:** 2020-09-30

**Authors:** Jan H. Overbeck, Werner Kremer, Remco Sprangers

**Affiliations:** Department of Biophysics I, Regensburg Center for Biochemistry, University of Regensburg, 93053 Regensburg, Germany

**Keywords:** Fluorine, Large complexes, Protein folding, Relaxation dispersion, Structural dynamics.

## Abstract

**Abstract:**

Proteins and nucleic acids are highly dynamic bio-molecules that can populate a variety of conformational states. NMR relaxation dispersion (RD) methods are uniquely suited to quantify the associated kinetic and thermodynamic parameters. Here, we present a consistent suite of ^19^F-based CPMG, on-resonance R_1ρ_ and off-resonance R_1ρ_ RD experiments. We validate these experiments by studying the unfolding transition of a 7.5 kDa cold shock protein. Furthermore we show that the ^19^F RD experiments are applicable to very large molecular machines by quantifying dynamics in the 360 kDa half-proteasome. Our approach significantly extends the timescale of chemical exchange that can be studied with ^19^F RD, adds robustness to the extraction of exchange parameters and can determine the absolute chemical shifts of excited states. Importantly, due to the simplicity of ^19^F NMR spectra, it is possible to record complete datasets within hours on samples that are of very low costs. This makes the presented experiments ideally suited to complement static structural information from cryo-EM and X-ray crystallography with insights into functionally relevant motions.

**Graphic abstract:**

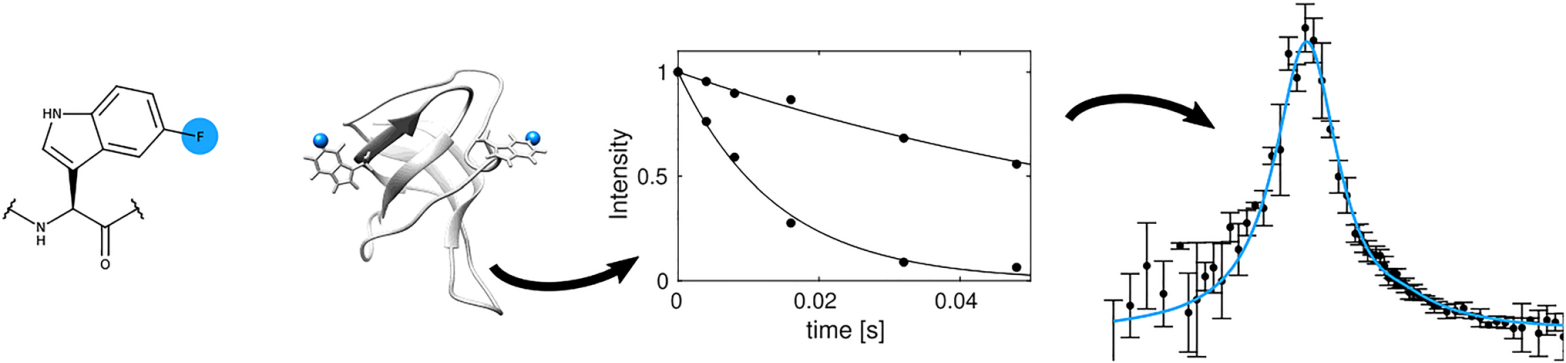

**Electronic supplementary material:**

The online version of this article (10.1007/s10858-020-00348-4) contains supplementary material, which is available to authorized users.

## Introduction

Bio-molecules are inherently dynamic and populate a number of structurally different states. NMR spectroscopy is a unique tool to experimentally investigate these bio-molecular motions with atomic resolution. When the exchange rates between the different states are on the millisecond timescale Carr–Purcell–Meiboom–Gill (CPMG) and rotating-frame relaxation experiments at different effective magnetic fields can be exploited to record relaxation dispersion (RD) profiles. These profiles directly depend on thermodynamic (populations) and kinetic (rates) parameters of the exchange process and structural information (chemical shifts) of sparsely populated (invisible), short-lived excited states can be obtained. For proteins under 20 kDa, RD experiments can be recorded on protonated ^15^N-labeled samples, whereas deuteration and transverse relaxation optimized spectroscopy (TROSY; Pervushin et al. 1997) are required for larger systems. In very large complexes (> 80 kDa), methyl group labeling in a fully deuterated background combined with methyl TROSY based approaches (Tugarinov et al. 2003; Schütz and Sprangers 2019; Abramov et al. 2020) can be used to record ^13^C single quantum (SQ; Skrynnikov et al. 2001; Lundström et al. 2007; Rennella et al. 2016) and ^13^C multiple quantum (MQ; Korzhnev et al. 2004) as well as ^1^H SQ (Tugarinov and Kay 2007; Baldwin et al. 2010; Otten et al. 2010; Weininger et al. 2012), double quantum (DQ; Gopalan et al. 2018) and triple quantum (TQ; Gopalan et al. 2018) RD profiles.

Recently, fluorine (^19^F) NMR has regained attraction (Hellmich et al. 2009; Liu et al. 2012; Kim et al. 2013; Kitevski-Leblanc et al. 2013; Aramini et al. 2014; Hoang and Prosser 2014; Manglik et al. 2015; Matei and Gronenborn 2016; Lu et al. 2019; Huang et al. 2020). This spin is absent from virtually all biomolecules, however, ^19^F probes can be artificially introduced into proteins by incorporation of fluorinated amino acids (Crowley et al. 2012) or through post-translational modification with fluorine-containing tags (Brauer and Sykes 1986). These ^19^F labeling strategies provide a number of advantages. First, the resulting samples contain only a limited number of NMR probes and spectra can often be recorded in a simple 1 dimensional (1D) manner. Second, in most cases, fluorine-based experiments can be recorded in a fully protonated background and samples can thus originate from sources where deuteration or methyl group labeling is not easily achievable (e.g. mammalian expression systems). Third, ^19^F experiments can complement and verify information from deuterated systems, where structure, stability and dynamics can be altered due to the pervasive isotopic substitution (Korzhnev et al. 2005a, b). Fourth, ^19^F shares favorable characteristics with ^1^H with respect to RD experiments (Juen et al. 2016), including reduced sample heating on probe heads with inverse coil configuration, short pulses and an extended range of accessible effective field strengths. Finally, the ^19^F chemical shift dispersion is large, which can result in significant chemical shift differences between the ground state and excited states and thus RD profiles that have large amplitudes.

In the past, ^19^F RD CPMG experiments have been exploited to study dynamic protein interfaces (Aramini et al. 2014), dynamic dimer asymmetry (Kim et al. 2017), allostery (Manglik et al. 2015), ligand-bound states (Hoang and Prosser 2014), protein folding (Kitevski-Leblanc et al. 2013) and fold-switching (Liebau et al. 2020). In those experiments, a varying number of 180° pulses is applied within a fixed relaxation delay such that exchange induced line-broadening can be suppressed. Fitting of these RD profiles with analytical or numerical approaches provides insight into the exchange constant k_ex_, the populations p_A_ and p_B_ as well as the absolute value of the chemical shift difference |Δω| between the two states. However, in many cases these data are not sufficient to unambiguously and accurately determine these parameters as significantly different exchange regimes can result in similar RD profiles. In those cases R_1ρ_ experiments can be used to resolve these ambiguities. In R_1ρ_ experiments the effective relaxation rate is measured in the rotating frame as a function of spin-lock field strengths (“on-resonance”) or as a function of spin-lock offsets (“off-resonance”). Compared to the CPMG approach, this has several advantages. First, R_1ρ_ experiments can access higher frequencies and thus allow quantification of faster dynamics. Second, off-resonance experiments can provide not only the magnitude but also the sign of Δω. Third, the sampling of points in the frequency dimension is not restricted, as it is in CPMG experiments where only frequencies that correspond to an integer number of 180° pulses in the relaxation delay are possible. Motivated by these considerations, we developed a suite of one-dimensional CPMG, on-resonance R_1ρ_ and off-resonance R_1ρ_ pulse sequences for ^19^F nuclei. We validate the experiments on the unfolding transition of a cold shock protein, show that they yield consistent exchange parameters and extract thermodynamic information from a temperature series of RD datasets. Moreover, we demonstrate the applicability of ^19^F rotating frame relaxation to a fully protonated 360 kDa protein complex.

## Materials and methods

### Molecular biology

The protein coding sequence of cold shock protein from *Thermotoga maritima* (NCBI reference sequence WP_004082199.1, hereafter referred to as TmCsp, internal reference: 2093) was codon optimized with COOL (http://bioinfo.bti.a-star.edu.sg/COOL/), synthesized by Integrated DNA Technologies (Coralville, USA) and cloned into a pETGB-1a vector (kindly provided by Dr. Arie Geerlof, Helmholtz Zentrum München). The vector contains an N-terminal His6-GB1-tag followed by a tobacco etch virus protease (TEV protease) cleavage site constituted by the amino acid sequence ENLYFQGG. The final protein sequence therefore contains an additional GG at the N-terminus of the protein.

### NMR sample preparation

#### *Thermotoga maritima* Csp

Plasmids were transformed into *Escherichia coli* BL21(DE3) CodonPlus-RIL cells (Stratagene) and grown in LB over night. Subsequently, M9 medium was inoculated with the LB culture and grown to an optical density OD_600_ of 1.0 at 37 °C. The medium was then supplemented with 50 mg/l 5-fluoroindole (Crowley et al. 2012) that was dissolved in DMSO at a 100 mg/ml stock concentration. 45–60 min later protein expression was induced by addition of 1 mM IPTG and the culture was shifted to 20 °C. Cells were harvested by centrifugation 12–18 h after induction. Cell pellets were resuspended in 10 mM Tris, pH 8.0, 10 mM imidazole, 1 mM EDTA, 1:1000 Triton X-100, 1 mg/l lysozyme and 0.2 U/ml DNaseI by vortexing for 30 min at 4 °C and then lysed by sonification. After addition of 5 mM MgCl_2_ and 5 µg/ml RNaseA the sample was incubated for 2 h at 37 °C. The cell debris was removed by centrifugation, the supernatant was additionally cleared with a 0.45 µm syringe filter and loaded onto a nickel–NTA gravity flow column that was pre-equilibrated with buffer A (10 mM Tris, 10 mM imidazole, pH 8.0). Subsequently, the column was washed with 20–30 column volumes of buffer A and the bound protein was eluted with 10 mM Tris, 300 mM imidazole, pH 8.0. The His_6_-GB1-tag was cleaved by addition of a His_6_-tagged TEV protease during dialysis for 12–18 h against 10 mM Tris, pH 8.0, 1 mM DTT at 20 °C. In order to separate the His-tagged proteins from the target protein, the dialyzed proteins were loaded on a nickel–NTA column pre-equilibrated with 10 mM Tris, pH 8.0, 10 mM imidazole. The column flow-through was dialyzed for 12–18 h against 10 mM NaHPO_4_, pH 6.8 and loaded onto a 5 ml Heparin HP HiTrap™ column. The protein was eluted with a salt gradient (0–500 mM NaCl in the presence of 1 mM EDTA) and the target fractions were combined and concentrated to a final volume of 1–1.5 ml. Finally, the protein was purified using a 16/600 Superdex S75 column in 50 mM Tris, pH 8.0, 100 mM NaCl, 1 mM EDTA. The target fractions were combined and concentrated with a simultaneous buffer exchange to 50 mM NaHPO_4_, pH 6.5, 20 mM NaCl, 0.2 mM EDTA, 1 mM DTT. The NMR sample was supplemented with 0.03% NaN_3_ and 5% D_2_O, the final protein concentration was 550 μM.

#### *Thermoplasma acidophilum* α_7_α_7_ complex

Plasmids were transformed into *E. coli* BL21(DE3) CodonPlus-RIL cells (Stratagene) and grown in LB over night. Fresh LB medium including antibiotics was inoculated from the overnight culture and grown to an optical density OD_600_ of 0.6–0.8 at 37 °C. The culture was shifted to 20 °C and protein expression induced with 0.5 mM IPTG for 12–18 h. Cell pellets were resuspended in 50 mM sodium phosphate, pH 7.4, 400 mM NaCl, 10 mM imidazole, 1 mM EDTA, 1:1000 Triton X-100, 1 mg/l lysozyme and 0.2 U/ml DNaseI by vortexing for 30 min at 4 °C and lysed by sonification. The cell debris was removed by centrifugation, the supernatant was additionally cleared with a 0.45 µm syringe filter and loaded on a nickel–NTA gravity flow column that was pre-equilibrated with buffer A2 (50 mM sodium phosphate, pH 7.4, 400 mM NaCl, 10 mM imidazole). In order to remove weakly bound contaminating proteins, the column was washed with 20–30 column volumes of buffer A2, after which the protein was eluted with 50 mM sodium phosphate, pH 7.4, 150 mM NaCl, 300 mM imidazole. The His_6_ tag was cleaved with His_6_-tagged TEV protease while dialyzing the elution against 50 mM sodium phosphate, pH 7.4, 150 mM NaCl, 1 mM DTT at 4 °C. The dialyzed sample was loaded on a second nickel–NTA column pre-equilibrated with buffer A2. The column flow-through was concentrated to 1 ml and finally purified using a 16/600 Superdex S200 column in 25 mM HEPES, pH 7.3, 125 mM NaCl, 1 mM DTT. The target fractions were combined and concentrated again to a volume of 0.5 ml. Bromotrifluoroacetone (Sigma-Aldrich) was added to a final concentration of 10 mM and incubated at 37 °C for 30′. The reaction was quenched with 20 mM DTT and the sample was purified over a PD 10 column pre-equilibrated with 25 mM HEPES, pH 7.3, 125 mM NaCl, 1 mM DTT. The NMR sample was supplemented with 0.03% NaN_3_ and 5% D_2_O, the final protein concentration was 1.3 mM (monomer concentration), corresponding to 93 µM α_7_α_7_ complex for the 18C mutant, and 0.75 mM (monomer concentration), corresponding to 54 µM α_7_α_7_ complex for the 35C mutant.

### NMR spectroscopy

**Fig. 1:**
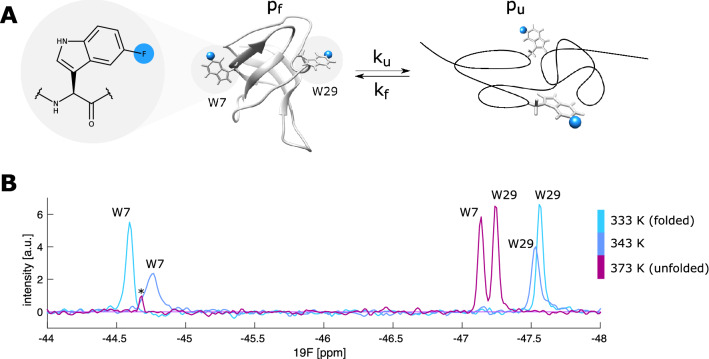
The folding-unfolding exchange of the TmCsp protein. **a** The chemical structure of 5-fluoro tryptophan (5FW; left) and a schematic presentation of the (un-) folding of TmCsp, which contains 5FW residues at positions 7 and 29 (Protein Data Bank, PDB ID 1G6P Kremer et al. 2001). **b** ^19^F spectra of TmCsp at 333 K, 343 K and 373 K with assignments for Trp7 and Trp29. The peak labeled with an asterisk results from a small impurity in the sample. This impurity resonates outside the displayed spectral window at the two lower temperatures

All NMR RD experiments were recorded on a 500 MHz Bruker NEO NMR spectrometer equipped with triple resonance TCI H/F–C–N–D nitrogen cooled probehead, where the proton coil was tuned and matched to the ^19^F resonance frequency (470 MHz). Data was acquired with 1024 points in the direct dimension and a relaxation delay of 1.5 s. The 1D spectra of TmCsp (Fig. 1) were recorded at 333 K, 343 K and 373 K on a Bruker 600 MHz spectrometer equipped with a TBI H–F–D probehead. Temperatures were calibrated using an ethylene glycol sample. Both the ethylene glycol sample as well as the sample of 5-fluoroindole dissolved in glycerol were locked using a capillary filled with D_2_O that was inserted into the 5 mm NMR tube. ^19^F spectra were referenced to TFA indirectly by measuring the absolute proton frequency of DSS and using a correction factor Ξ of 0.940866982 (Maurer and Kalbitzer 1996).

The CPMG experiments for TmCsp were recorded with a relaxation delay of 40 ms and with 23 different CPMG frequencies of 50, 100, 150, 200, 250, 300, 350, 400, 450, 500, 600, 700, 800, 900, 1000, 1500, 2000, 2500, 3000, 3500, 4000, 4500 and 5000 Hz.

The CPMG experiments for the α_7_α_7_ complex at 303 K, 313 K and 323 K were recorded with a relaxation delay of 8 ms and with 22 different CPMG frequencies of 125, 250, 375, 500, 625, 750 875, 1000, 1125, 1250, 1500, 1750, 2000, 2250, 2500, 2750, 3000, 3250, 3500, 4000, 4500 and 5000 Hz; the experiments at 293 K were recorded with a relaxation delay of 4 ms and with 20 different CPMG frequencies of 250, 500, 750, 1000, 1250, 1500, 1750, 2000, 2250, 2500, 2750, 3000, 3250, 3500, 3750, 4000, 4250, 4500, 4750 and 5000 Hz.

**Fig. 2:**
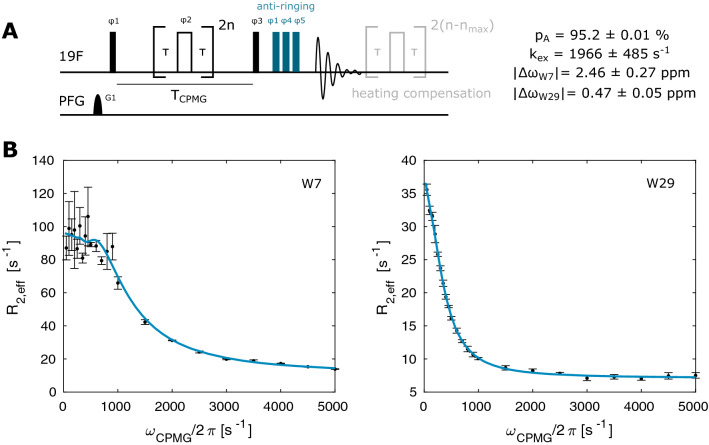
^19^F CPMG experiment. **a** Pulse sequence for recording ^19^F CPMG RD profiles. Narrow (wide) rectangles indicate 90° (180°) pulses, which are applied along the *x-*axis unless indicated otherwise. The phase cycle is φ1 = φ_rec_ = [x, x, − x, − x, y, y, − y, − y], φ2 = [y, − y, y, − y, x, − x, x, − x]. The number of CPMG pulses applied during the CPMG time T_CPMG_ is given by 2n, where n is an integer. The maximum number n_max_ is chosen so that the highest applied frequency υ_CPMG_ = n_max_/T_CPMG_ is at or below 5 kHz. **b** CPMG relaxation dispersion profiles for W7 and W29 recorded at 344 K. The size of the error-bars correspond to 1 standard deviation

The fully extended phase cycle for the ^19^F CPMG including the aring-sequence (Fig. 2a) is φ1 = [0 0 0 0 0 0 0 0 0 0 0 0 0 0 0 0 2 2 2 2 2 2 2 2 2 2 2 2 2 2 2 2 1 1 1 1 1 1 1 1 1 1 1 1 1 1 1 1 3 3 3 3 3 3 3 3 3 3 3 3 3 3 3 3], φ2 = [1 1 1 1 1 1 1 1 3 3 3 3 3 3 3 3 1 1 1 1 1 1 1 1 3 3 3 3 3 3 3 3 0 0 0 0 0 0 0 0 2 2 2 2 2 2 2 2 0 0 0 0 0 0 0 0 2 2 2 2 2 2 2 2], φ3 = [2 2 2 2 2 2 2 2 2 2 2 2 2 2 2 2 0 0 0 0 0 0 0 0 0 0 0 0 0 0 0 0 3 3 3 3 3 3 3 3 3 3 3 3 3 3 3 3 1 1 1 1 1 1 1 1 1 1 1 1 1 1 1 1], φ4 = [2 0 2 0 2 0 2 0 2 0 2 0 2 0 2 0 0 2 0 2 0 2 0 2 0 2 0 2 0 2 0 2 3 1 3 1 3 1 3 1 3 1 3 1 3 1 3 1 1 3 1 3 1 3 1 3 1 3 1 3 1 3 1 3], φ5 = [0 0 2 2 1 1 3 3 0 0 2 2 1 1 3 3 2 2 0 0 3 3 1 1 2 2 0 0 3 3 1 1 1 1 3 3 2 2 0 0 1 1 3 3 2 2 0 0 3 3 1 1 0 0 2 2 3 3 1 1 0 0 2 2], φ_rec_= [0 2 2 0 1 3 3 1 0 2 2 0 1 3 3 1 2 0 0 2 3 1 1 3 2 0 0 2 3 1 1 3 1 3 3 1 2 0 0 2 1 3 3 1 2 0 0 2 3 1 1 3 0 2 2 0 3 1 1 3 0 2 2 0], where [0, 1, 2, 3] corresponds to [x, y, − x, − y].

The R_1ρ_ on-resonance experiments for TmCsp were based on six different spin-lock times T_SL_ (0, 4, 8, 16, 32 and 48 ms) and 28 different spin lock fields (351.05, 393.88, 441.94, 495.87, 556.37, 624.26, 700.43, 785.9, 881.79, 989.38, 1110.11, 1245.56, 1397.54, 1568.07, 1759.4, 1974.08, 2214.95, 2485.22, 2788.46, 3128.71, 3510.47, 3938.81, 4419.42, 4958.67, 5563.72, 6242.59, 7004.3, 7858.96 Hz). The ^19^F frequency carrier was centered on the respective peak maximum in the 1D ^19^F spectrum.

The R_1ρ_ on-resonance experiments for the α_7_α_7_ complex were based on six different spin-lock times T_SL_ (0, 2, 4, 8, 12 and 16 ms) and 31 different spin lock fields (103.54, 116.17, 130.35, 146.25, 164.10, 184.12, 206.591, 231.80, 260.08, 291.82, 327.42, 367.37, 412.20, 462.50, 518.93, 582.25, 653.29, 733.01, 822.45, 922.80, 1035.40, 1161.74, 1641.00, 2600.81, 3274.23, 4122.01, 5189.30, 5822.49, 6532.94, 7330.08, 8224.49 Hz). The ^19^F frequency carrier was centered on the respective peak maximum in the 1D ^19^F spectrum.

The R_1ρ_ off-resonance experiments for TmCsp were based on six different spin-lock times T_SL_ (0, 4, 8, 16, 32 and 48 ms), 71 different offsets frequencies (+ and − 0, 50, 100, 150, 200, 250, 300, 350, 400, 450, 500, 600, 700, 800, 900, 1000, 1100, 1200, 1300, 1400, 1500, 1600, 1700, 1800, 1900, 2000, 2100, 2200, 2300, 2400, 2500, 2600, 2800, 3000, 3200 and 3400 Hz) and 4 different spinlock fields (100, 200, 300 and 400 Hz). The spin-lock is flanked by two hard pulses that transfer the magnetization to the spin-lock angle θ. θ is calculated from the spin-lock power and the spin-lock offset as θ = tan^−1^(ω_SL_/ΔΩ), after which the length of the θ-hard pulse is calculated from the 90° hard pulse p_hard_ as pθ = θ/90° * p_hard_. The ^19^F frequency carrier was centered on the Trp7 peak maximum in the 1D ^19^F spectrum.

All relaxation dispersion datasets were recorded in triplicate in order to obtain the indicated error estimates.

#### Data processing/analysis/fitting

NMR experiments were processed with the NMRpipe software (Delaglio et al. 1995) and analyzed with in house written Python and Matlab scripts.

#### CPMG

Effective transverse relaxation rates were calculated as R^eff^_2_ = − ln(I/I_0_)/T_CPMG_, where I is the peak intensity, I_0_ is the reference intensity recorded without the CPMG element and T_CPMG_ is the constant CPMG time. The CPMG profiles were numerically fitted (Korzhnev et al. 2004) to a two-state model using an in-house Matlab script. The transverse relaxation rates of the folded state and the unfolded state were assumed to be equal in the absence of exchange (R_2,f_ = R_2,u_).

#### On-resonance R_1ρ_

R_1ρ_ rates were extracted from an exponential fit of the resonance intensities in experiments with six different T_SL_ times. R_1ρ_ data was fitted with an in-house Matlab script using a numerical approach in which R_1ρ_ is approximated as the least negative eigenvalue of the Bloch–McConnell evolution matrix (Trott and Palmer 2002).

#### Off-resonance R_1ρ_

R_1ρ_ rates were extracted from an exponential fit of the resonance intensities in experiments with six different T_SL_ times. The off-resonance R_1ρ_ data was fitted with an in-house Matlab script using the Laguerre approximation as an analytical equation that is valid for off-resonance R_1ρ_ data (Koss et al. 2017; Miloushev and Palmer 2005).

#### Monte Carlo simulations

Standard deviations and mean values of all datapoints were calculated using the experimental triplicates. In order to assess the uncertainties from the fitting, we performed a Monte Carlo Simulation with 100 fit iterations, where all data-points were varied in each iteration according to a Gaussian distribution.

#### Thermodynamic and kinetic analysis

Solutions for k_ex_ (= k_F_ + k_U_) and p_F_ from the CPMG fits at different temperatures were used to calculate the unfolding rate k_U_, the folding rate k_F_ and the equilibrium constant K_eq_ according to

1 $${p}_{U}=1-{p}_{F},$$

2 $${k}_{U}={p}_{U}\bullet {k}_{ex},$$

3 $${k}_{F}={p}_{F}\bullet {k}_{ex},$$

4 $${K}_{eq}={k}_{U}/{k}_{F}.$$

To obtain the thermodynamic parameters, first *K*_*eq*_ was fit according to

5 $${K}_{eq}={e}^{-{\Delta }G/RT}={e}^{(T{\Delta }S-{\Delta }H)/RT}.$$

The values obtained for the change in entropy ΔS and in enthalpy ΔH were subsequently used as constraints

6 $${\Delta }S={{\Delta }S}^{\ddagger F\to U}+ {\varDelta S}^{\ddagger U\to F},$$

7 $$\varDelta H={\varDelta H}^{\ddagger F\to U}+ {\varDelta H}^{\ddagger U\to F}.$$

in the fit equations

8 $${k}_{U}=\frac{1}{\tau _{TPT}}{e}^{({T{\Delta }S}^{\ddagger F\to U}-{\varDelta H}^{\ddagger F\to U})/RT},$$

9 $${k}_{F}=\frac{1}{\tau _{TPT}}{e}^{({T\varDelta S}^{\ddagger U\to F}-{\varDelta H}^{\ddagger U\to F})/RT},$$

where τ_TPT_ is the transition path time, ΔS and ΔH are the differences in entropy and enthalpy between the folded and the unfolded state, ${\varDelta S}^{\ddagger F\to U}$and ${\varDelta H}^{\ddagger F\to U}$ are the entropy and enthalpy differences between the folded state and the transition state and ${\varDelta S}^{\ddagger U\to F}$ and ${\varDelta H}^{\ddagger U\to F}$ are the entropy and enthalpy differences between the unfolded state and the transition state.

## Results

Here we introduce a suite of one-dimensional ^19^F pulse sequences for the collection of CPMG, on-resonance R_1ρ_ and off-resonance R_1ρ_ data that only require a limited amount of experimental time. We use the cold shock protein from *T. maritima* (TmCsp) to highlight the applicability of these sequences to accurately determine exchange parameters in biomolecules. TmCsp is a small (7.5 kDa) thermostable protein that undergoes an unfolding transition with a melting temperature of T_m_ = 359 K (Schuler et al. 2002a, b). Two ^19^F probes were introduced into the protein by replacing the two natural tryptophan residues (W7 and W29) with 5-fluoro tryptophan (5FW; Fig. 1a; Supporting Information). Both the natural and the 5FW-labeled protein follow a two state unfolding transition (Perl et al. 1998; Wassenberg et al. 1999; Schuler et al. 2002a, b), which makes it a well-suited system to establish our ^19^F RD experiments.

The ^19^F NMR spectra of the protein show two sharp resonances at low (333 K) and high (373 K) temperatures corresponding to the fully folded (light blue) and fully unfolded (red) states (Fig. 1b). Based on these spectra, the chemical shift differences between the folded and thermally unfolded protein are 2.53 ppm (W7) and 0.33 ppm (W29) respectively. Of note, at temperatures above 373 K the resonances of the unfolded state (red) continue to shift towards lower ppm values. Based on this temperature dependence of the unfolded state chemical shift we can derive that the chemical shift differences at 333 K are 2.35 ppm (W7) and 0.48 ppm (W29). At 343 K (blue) extensive line broadening is observed, which arises from the exchange (k_ex_ = k_F_ + k_U_) between the folded (F) and unfolded (U) states of the protein (Fig. 1a).

To quantify this exchange process we first recorded ^19^F CPMG RD profiles using the pulse sequence displayed in Fig. 2a (Supporting Information). This sequence contains two elements that we found to be particularly important for obtaining high quality data. First, we included a heat compensation block such that the sample is exposed to the same total RF power independent of the CPMG frequency. This is especially important when higher ^19^F CPMG frequencies (5 kHz) are used in combination with longer (> 20 ms) CPMG times. Second, when using a probehead where the ^1^H coil is de-tuned to record ^19^F spectra, we found broad baseline artifacts at the on-resonance ^19^F frequency that are due to suboptimal performance of the ^1^H RF coil for ^19^F experiments. These imperfections can be suppressed through the use of an “anti-ring” sequence (Gerothanassis 1987) that consists of three 90° pulses of different phases in combination with extensive phase cycling. We then used a sample that contains 5-fluoroindole in glycerol to show that the CPMG experiment results in flat RD profiles for a system that does not undergo chemical exchange (Fig. S1). Due to the high viscosity, the ^19^F T_1_ and T_2_ times are comparable to the ^19^F relaxation times in proteins making this a good testing system for biological samples.

Based on the above, we recorded CPMG RD profiles on TmCsp at 11.7 T (500 MHz ^1^H frequency) and 344 K, which is 15 K below the melting temperature of the protein. In total, we recorded a full dataset with 23 CPMG frequencies in approximately 40 min (64 scans, 1.5 s interscan delay), highlighting the efficiency of the 1D based ^19^F CPMG method. The ^19^F signals of the two ^19^F tryptophan residues show large dispersions, indicative of chemical exchange (Fig. 2b). A global numerical fit (solid line) according to a two-site exchange model (Supporting Information) gives an exchange rate of k_ex_ = 1966 ± 485 s^−1^, populations of p_F_ = 95.2 ± 0.1%, p_U_ = 4.8 ± 0.1% and chemical shift differences of |Δω_W7_| = 2.46 ± 0.27 ppm and |Δω_W29_| = 0.47 ± 0.05 ppm, where the errors in the extracted parameters are standard deviations calculated by Monte Carlo simulations based on three separately recorded datasets (Fig. S2). It is noteworthy that the determined chemical shift differences agree well with the values obtained from 1D spectra recorded at different temperatures (2.35 ppm and 0.48 ppm for W7 and W29 respectively; Fig. 1b), especially when considering that only two peaks at a single field strength are used to fit the exchange parameters. The relatively high accuracy of the extracted parameters can be explained based on the fact, that the values of |Δω| are significantly different for the two residues. The ratio between the kinetic parameter k_ex_ (1966 s^−1^) and the absolute value of |Δω|, which defines the exchange regime, effectively yields an intermediate exchange for W7 (|Δω_W7_| = 2.44 ppm = 2π·1170 Hz), but is in fast exchange for W29 (|Δω_W29_| = 0.46 ppm = 2π·216 Hz). Our data here show, that the exchange parameters can be reliably extracted from the CPMG dispersions alone. The associated standard deviations in the extracted parameters are nevertheless considerable (Fig. S2).

**Fig. 3:**
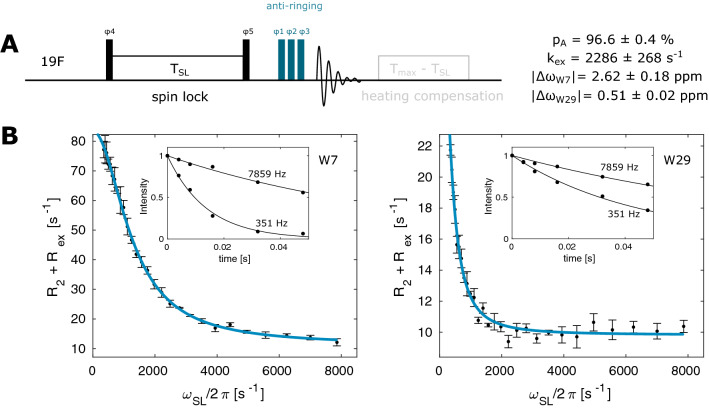
^19^F on-resonance R_1ρ_ experiment. **a** Pulse sequence for the ^19^F on-resonance R_1ρ_ experiment. Narrow (wide) rectangles indicate 90° (180°) pulses, which are applied along the x-axis unless indicated otherwise. The phase cycle is φ1 = x, φ2 = [− x, x], φ3 = [x, x, − x, − x, y, y, − y, − y], φ4 = [y], φ5 = [− y], φ_rec_ = [x, − x, − x, x, y, − y, − y, y]. **b** On-resonance R_1ρ_ relaxation dispersion profiles for W7 and W29. The size of the error-bars correspond to 1 standard deviation

To complement the CPMG RD data and to be able to study systems that exchange on faster timescales we made use of a ^19^F on-resonance R_1ρ_ pulse sequence (Fig. 3a and Supporting Information). We initially implemented the option to use adiabatic ramps with tanh/tan amplitude/phase modulation for the magnetization transfer to the transverse plane (Mulder et al. 1998). However, we found a transfer with hard pulses more convenient, as it is compatible with lower spin-lock powers, for which the adiabatic condition would otherwise be violated. As for the CPMG RD experiment, we observed significant baseline distortions in the spectra that result from acoustic ringing in the ^1^H/^19^F-coil at the ^19^F resonance frequency. These artifacts could be efficiently eliminated with an anti-ringing element. In addition, the pulse sequence includes a heating compensation for different spin-lock times T_SL_ to ensure a constant RF power. We recorded separate datasets for W7 and W29 at 11.7 T and 344 K with the carrier frequency placed on-resonance (either at W7 or at W29), each with 28 spin-lock field strengths between 0.35 kHz and 7.9 kHz. Every datapoint corresponds to an R_1ρ_ = R_2_ + R_ex_ value that is obtained by fitting of an exponential decay recorded over six time points T_SL_. The total experimental time for 1 complete R_1ρ_ dataset (32 scans, 1.5 s interscan delay) is around 2.3 h. To extract a reliable estimate of the error, we recorded three datasets for W7 and three datasets for W29. The data was simultaneously fitted using Trott’s and Palmer’s approach (2002) (solid line) and yields an exchange rate of k_ex_ = 2286 ± 268 s^−1^, populations of p_F_ = 96.6 ± 0.4%, p_U_ = 3.4 ± 0.4% and chemical shift differences of |Δω_W7_| = 2.62 ± 0.18 ppm and |Δω_W29_| = 0.51 ± 0.02 ppm. The extracted parameters agree well with the parameters that we extracted from the RD CPMG experiments (Fig. 2b) and with the chemical shift differences that we observe in the 1D NMR spectra (Fig. 1b).

It is noteworthy, that higher spin-lock fields up to 20 kHz have been employed in proton R_1ρ_ experiments on cryoprobes (Steiner et al. 2016), which accordingly expands the range of dynamics that can be studied with the presented fluorine R_1ρ_. On the other end, the lower limit of the spinlock field is dictated by the largest J-couplings present in the spin system (Zhao et al. 2014). In 5FW, the ^3^J_HF_ coupling of ~ 9.9 Hz restricts the use of spin-lock to fields below 30 Hz (Zhao et al. 2014). This limitation is eluded by the use of a dual ^1^H–^19^F probehead where proton-fluor couplings can be decoupled and is also not applicable in fluorine labels where no vicinal protons are coupled to the ^19^F spin, as is the case in bromotrifluoroacetone (BTFA; see below). We note that in the CPMG experiment the ^3^J_HF_ coupling leads to the interconversion of ^19^F in-phase and anti-phase magnetization during the periods 2τ between the refocusing pulses. Dependent on the CPMG frequency and on the difference of in-phase and anti-phase transverse relaxation rates, this can result in a distorted RD profile (Kay et al. 1992). This can be overcome by employing explicit relaxation–compensation, where in-phase and anti-phase contributions are averaged for all CPMG frequencies (Loria et al. 1999). However, in biological application of ^19^F NMR this problem is negligible for most practical purposes, as the anti-phase contributions only become relevant, when the periods 2τ between the refocusing pulses become longer than 1/4J. Even in the case of a strong three-bond H–F coupling of 10 Hz, this issue thus only arises for unusually long CPMG times (> 50 ms when 2 refocussing π pulses are used). Finally, it is worth mentioning that we here sampled six points of the exponential decay in order to rule out a non-exponential behavior of the spin-locked relaxation, that can arise due to inaccurate alignment of the magnetization with the spin lock field. The experimental time can be reduced at least by a factor of 3 when only a reference and a single timepoint are measured to determine the exponential decay for each spinlock field (Fig. S7), without a significant change in the values of the extracted parameters.

**Fig. 4:**
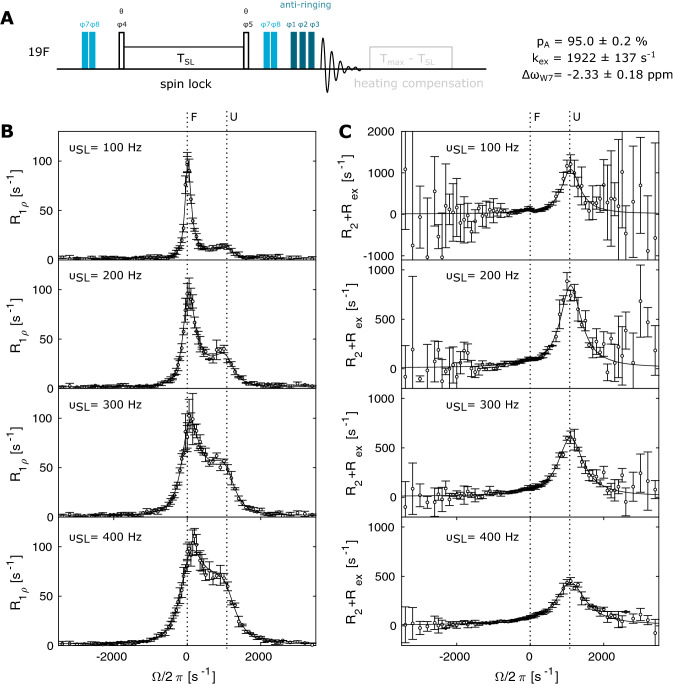
^19^F off-resonance R_1ρ_ experiment. **a** Pulse sequence for the ^19^F off-resonance R_1ρ_ experiment. Narrow rectangles indicate 90° pulses. The phase cycle is φ1 = x, φ2 = [− x, − x, x, x], φ3 = [x, x, x, x, − x, − x, − x, − x, y, y, y, y, − y, − y, − y, − y], φ4 = [y, − y], φ5 = [− y, y], φ6 = [x, − x], φ7 = [x], φ8 = [x, − x], φ_rec_ = [x, − x, − x, x, y, − y, − y, y]. The flip-angle of pulses that flank the spinlock block is θ, which ensures that the magnetization is aligned at the angle of the effective magnetic field. This angle depends on the offset and the spinlock power. The pulse pairs with phases φ7/φ8 are used to cycle the magnetization to ± z before the spin lock period and back to + z after the spin lock, which ensures that the rotating frame relaxation is symmetrically measured both above and below the transverse plane at all offsets. **b** R_1ρ_ off-resonance relaxation dispersion profiles and **c** corresponding R_2_ + R_ex_ contributions for W7 at spin-lock fields of 100 Hz, 200 Hz, 300 Hz and 400 Hz. Solid lines show the best fit to a two-state Laguerre approximation. The offsets of the folded (F) and the unfolded (U) state are indicated with dotted lines. Error bars show experimental uncertainty (1 standard deviation)

The CPMG and R_1ρ_ RD experiments only yield the absolute value of the chemical shift difference between the two states (|Δω_i_|). ^19^F off-resonance R_1ρ_ experiments (Fig. 4a and Supporting Information) on the other hand can be used to extract a full set of parameters (p_F_, k_ex_, R_2,i_, Δω_i_) from measurements at a single B_0_ field in case of a two-state exchange process (Trott and Palmer 2002; Korzhnev et al. 2005a, b). In that experiment the effective field, which is defined by the vector sum of the spin-lock field ω_1_ and the spin-lock offset ω_offset_, is now inclined by an angle θ. The magnetization is transferred from the ± z axis to an angle θ by hard pulses of the appropriate length, which allows to use very low spin-lock fields. Again, our pulse sequence included a heating compensation for the different spin lock lengths as well as an anti-ringing element. We recorded datasets for W7 at 11.7 T and 344 K at spin-lock fields of 100 Hz, 200 Hz, 300 Hz and 400 Hz and with 71 offsets in each experiment. The total experimental time for this complete dataset (16 scans, interscan delay 1.5 s) is 11.5 h. To estimate the error in the data, this dataset was recorded three times. Figure 4b shows the R_1ρ_ data together with a global fit of all off-resonance datasets (solid line). A main peak corresponding to the observable resonance (F) is centered at zero offset, with a width determined by the spin lock power ω_1_. Exchange between the folded and the unfolded state leads to the appearance of a second peak that is centered around the resonance frequency of the excited, unfolded state (U). At 100 and 200 Hz, this peak gives rise to a distinct maximum; at higher spin-lock frequencies the resonances merge, but still add up to a clearly asymmetric profile that is indicative of the exchange process. The contribution of R_2_ + R_ex_ can be extracted from the R_1ρ_ data as R_2_ + R_ex_ = (R_1ρ_ – R_1 _* cos^2^θ)/sin^2^θ (Fig. 4c). The error in R_2_ + R_ex_ rapidly increases with the spin-lock offset, because the contribution is scaled with the inverse of sin^2^θ. At the same time, the error at a given offset decreases when increasing the spin lock power, as is readily visible from the data between 100 and 400 Hz power. Based on a global fit of the complete off-resonance dataset, we extract an offset Δω_W7_ of − 2π·1097 ± 84 Hz = − 2.33 ± 0.18 ppm, a ground state population of p_F_ = 95.0 ± 0.2% and an exchange constant of k_ex_ = 1922 ± 137 s^−1^, which is close to the values obtained by the CPMG and on-resonance R_1ρ_ RD experiments. We also observe that the single datasets at 100 Hz or 200 Hz can be used to obtain accurate exchange parameters. The 300 Hz data, and certainly the 400 Hz data, on the other hand fail to give well-defined results (Fig. S4), because a higher spin lock field increasingly overwrites off-resonance effects (Trott and Palmer 2002).

In order to evaluate the consistency between the CPMG, the on-resonance R_1ρ_ and the off-resonance R_1ρ_ experiments, we used a global fit for all datasets (Figs. S5, S6). We expect that a global fit yields the most precise parameters, because the three experiments contain redundant information about the protein folding/unfolding transition. In the fit, we optimized the following parameters: k_ex_, Δω_W7_, Δω_W29_, p_F_ and R_2_. Because we observed small deviations on the order of 2 Hz in the R_2_ values from the CPMG and the R_1ρ_ experiments we introduced two independent R_2_ variables: R_2_^CPMG^ and R_2_^R^^1ρ^. Based on that, we obtained a ground state population of p_F_ = 94.8 ± 0.1%, an exchange constant of k_ex_ = 1737 ± 54 s^−1^, and chemical shift differences of Δω_W7_ = 2π·1086 ± 66 Hz  = −2.31 ± 0.14 ppm, |Δω_W29_| =  2π·202 ± 5 Hz = 0.43 ± 0.01 ppm. These parameters are in agreement with the individually fitted datasets, but have a significantly increased precision (Fig. S5).

**Fig. 5:**
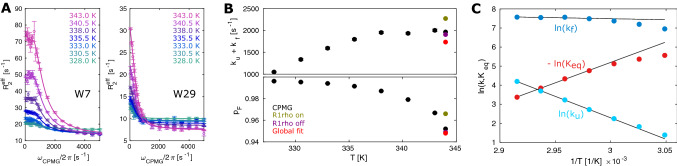
Kinetic and thermodynamic analysis of the TmCsp folding. **a** CPMG curves with a global fit, assuming a constant chemical shift difference between the folded and the unfolded state across all temperatures. **b** Temperature dependence of exchange rates and populations from 328 to 343 K. For comparison, the results from the experiments at 344 K are included. **c** Temperature dependence of k_u_, k_f_ and K_eq_ of 5FW-labeled TmCsp from 328 to 343 K. Note that only the data between 335.5 and 343 K was used to extract the thermodynamic parameters

Temperature dependent RD measurements are well suited to extract thermodynamic parameters of chemical exchange processes (Mulder et al. 2001; Nikolova et al. 2011; Audin et al. 2013). Here, we measured ^19^F CPMG RD profiles at seven different temperatures between 328 and 343 K and determined the unfolding and folding rates (k_U_, k_F_) as well as equilibrium constants K_eq_ = k_U_/k_F_ (Fig. 5; Supporting Information). The temperature-profile of K_eq_ between 335.5 and 343 K was subsequently fit to an Arrhenius model for Gibbs free energy ΔG = ΔH − TΔS = − RT ln K_eq_, from which we obtain ΔH = 43.9 ± 1.0 kcal/mol and TΔS = 40.6 ± 1.0 kcal/mol at 343 K, indicating an entropy–enthalpy compensation during TmCsp unfolding. Similarly, transition state theory can be used to obtain estimates of the enthalpic and entropic contributions to the energy barrier that separates the folded and the unfolded state. Because the traditional pre-exponential factor k_B_T/h in the Eyring equation is on the timescale of covalent bond vibrations and hence a suboptimal estimate for large-scale biomolecular dynamics (Moore 2012; Vallurupalli et al. 2016), we chose to estimate it as 1/τ_TPT_, where τ_TPT_ is the transition path time. Based on MD simulations and single-molecule fluorescence studies that show that τ_TPT_ is similar even for proteins with vastly different folding rates (Shaw et al. 2010; Chung et al. 2012; Vallurupalli et al. 2016), we assumed τ_TPT_ to be 1 µs and obtained values of ΔH^‡^ = 45.8 ± 1.2 kcal/mol and TΔS^‡^ = 39.2 ± 1.2 kcal/mol at 343 K. We note that the value of the transition path time hardly effects the enthalpy of the transition state, but the entropic contribution is dependent on it and varies from 37.7 ± 1.1 kcal/mol for τ_TPT_ = 0.1 µs to 40.9 ± 1.3 kcal/mol for τ_TPT_ = 10 µs.

Based on these findings a higher temperature stabilizes the unfolded state (p_U_=1 − p_F_; Fig. 5b, lower panel; Fig. 5c, red curve; ΔG_F→U_ decreases from 3.29 to 2.32 kcal/mol between 335.5 and 343 K) due to an entropic benefit. Likewise, but to a slightly lesser extent, a higher temperature reduces the free energy of the transition state. As a result, an increase in temperature results in a decreased energy barrier for the unfolding reaction (ΔG^‡^_F→U_ decreases from 9.07 to 8.19 kcal/mol between 335.5 and 343 K), while the energy barrier for folding is slightly increased, (ΔG^‡^_U→F_ changes from 5.78 to 5.87 kcal/mol between 335.5 and 343 K). This leads to the counterintuitive situation where the exchange rate (k_ex_ = k_F_ + k_U_ = exp(− ΔG/RT) that is dominated by k_F_) remains nearly constant with temperature (Fig. 5b, top panel), because ΔG^‡^_U→F_ and T increase at approximately the same pace.

**Fig. 6:**
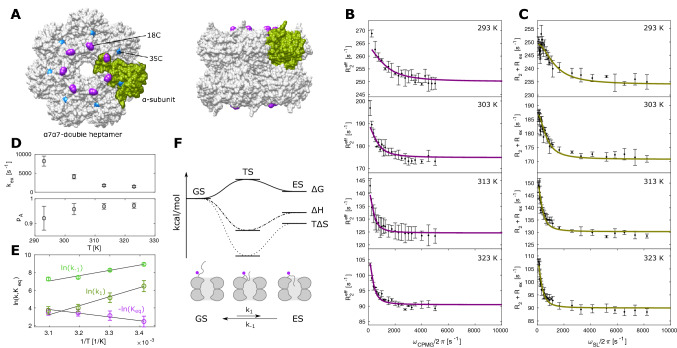
^19^F Relaxation dispersion experiments of the 360 kDa α_7_α_7_ double heptamer. **a** Model of the double heptameric α_7_α_7_ complexes with 14 BTFA labeling sites at position 18C (purple) of each subunit. Position 35 that does not show exchange (Fig. S8) is indicated in cyan. The model of the complex is based on the structure of the 20S proteasome from *T. acidophilum* (PDB ID 1PMA). **b** CPMG experiments from 293 to 323 K. **c** On-resonance R_1ρ_ experiments from 293 to 323 K. **d** Plot of exchange rates against temperatures as derived from the global fit of all RD data. **e** Logarithmic plot of kinetic rates against inverse temperature. **f** Schematic presentation of ΔG, ΔH and TΔS changes from the ground state GS to the excited state ES via a transition state TS

For high molecular weight systems, the R_2_ relaxation rates will be significantly higher than the rates that we observed for TmCsp. In those cases, the CPMG relaxation time has to be shortened to such an extent, that the lower relaxation dispersion frequencies can no longer be sampled, which will hamper the faithful examination of dynamics. This limitation can be resolved with R_1ρ_ experiments that are able to sample arbitrary frequencies between a lower limit given by scalar couplings and an upper limit given by the probe head power limits. To illustrate this we applied ^19^F RD experiments to a 360 kDa double heptameric α_7_α_7_ complex (half proteasome) derived from the α_7_β_7_β_7_α_7_ 20S proteasome of *T. acidophilum* (Fig. 6). The seven N-termini in each of the two α-rings have been shown to sample conformations in- and outside of the ring pore, thereby forming a functionally important dynamic gate (Sprangers and Kay 2007; Religa et al. 2010). We introduced single cysteine mutations at positions 18 and 35 of the α-subunit and labeled the purified half proteasome with BTFA to introduce a site specific ^19^F probe. The sample with a cysteine at position 35, that is remote from the pore, showed a modest relaxation rate of 122 Hz and a flat CPMG curve (Fig. S8). However, for the 18C-sample, we found that the single fluorine resonance at − 84 ppm shows strongly temperature dependent peak widths between 161 Hz (293 K) and 88 Hz (323 K). To monitor the gate dynamics, we collected CPMG (Fig. 6b) and R_1ρ_ (Fig. 6c) datasets at 293 K, 303 K, 313 K and 323 K. The constant time in the CPMG experiment had to be restricted to 4 ms at 293 K which results in CPMG frequencies that are multiples of 250 Hz. In the rotating frame relaxation experiments, on the other hand, we recorded 8 different R_1ρ_ rates below 250 Hz, which provides essential information on the exchange process. Of note, the trifluoroacetone moiety lacks vicinal protons that couple to the ^19^F spins and the largest scalar coupling present is a ^4^J_FH_ coupling of around 1 Hz (Abraham et al. 1996) which eliminates the lower bound for the sampling of the frequencies. To extract exchange parameters, we simultaneously fitted the CPMG and R_1ρ_ data at all temperatures with a two-state model, assuming that the Δω between the ground state and the excited state is independent of the experimental temperature. Based on that, we unexpectedly observe that higher temperatures result in significantly slower exchange rates and increased populations of the ground state (Fig. 6d). In line with this, an Eyring plot of the rate constants k_1_ and k_−1_ shows a linear decrease in ln(k) for both constants (Fig. 6e). These results arise from a negative entropy of the excited state (TΔS = − 10.1 ± 3.0 kcal/mol at 313 K) as well as the transition state (TΔS^‡^ = − 26.1 ± 4.8 kcal/mol at 313 K), which makes the excited state unfavorable at higher temperatures (Fig. 6f). Structurally, this behavior can be explained by a model where the N-terminal extensions are flexible in the open ground state and motionally restricted in the excited state where this region of the protein is located within the annulus pore (Fig. 6f).

## Discussion and conclusions

Taken together, we here present a suite of one-dimensional ^19^F RD experiments, to study exchange processes in ^19^F-labeled proteins. The on-resonance and off-resonance R_1ρ_ pulse sequences provide a number of important advantages over the complementary CPMG RD experiments. First, the rotating frame experiments can make use of very low spin lock fields, opening the possibility to obtain a full set of parameters at a single static magnetic field. The ability to sample arbitrary frequencies also allows study cases where the ^19^F signals experience fast relaxation and where CPMG experiments are restricted to higher frequencies due to the requirement for very short (2–3 ms) CPMG times (Manglik et al. 2015). In that light, it is also worth mentioning that short R_1ρ_ sequences could be combined with recently introduced two-dimensional (2D) ^19^F–^13^C TROSY experiments, where destructive interferences between DD and CSA mechanisms in aromatic ^19^F–^13^C groups result in improved ^13^C line widths (Boeszoermenyi et al. 2019). Second, compared to CPMG RD experiments, the rotating frame approaches can expand the timescale of chemical exchange that is accessible to very fast (µs timescale) processes. Third, the off-resonance R_1ρ_ experiments provide important information on the sign of the chemical shift differences, which provides direct information on the nature of the excited state.

We anticipate that fluorine NMR methods will become increasingly popular. Indeed, studies in the recent years have shown first examples of its applicability to challenging systems including GPCRs, where the possibilities for protein deuteration and methyl group labeling are limited due to the requirements of eukaryotic expression systems. Proteins from these sources can, however, be labeled with trifluoromethyl groups in a straightforward posttranscriptional manner at very low costs. The very short experimental times of the ^19^F based 1D experiments will further increase the applicability to systems that are not stable over time and where time intensive ^13^C, ^15^N or ^1^H based RD are not feasible. In the case of the TmCsp, the use of ^19^F RD approaches was motivated by enhanced amide-proton exchange rates at our experimental conditions, which resulted in very low quality ^1^H–^15^N based experiments. In general, ^19^F based experiment can thus be expected to outperform more traditional measurements at the elevated temperatures or at a higher pH. Finally, recent advances in labeling techniques have made RNA and DNA accessible for ^19^F NMR (Sochor et al. 2016; Nußbaumer et al. 2020; Baranowski et al. 2020), thereby further expanding the applicability of the presented methodologies. In summary, our approach will strengthen the use of ^19^F NMR to accurately quantify dynamic processes in a broad range of biological systems. This applicability of this strategy will benefit from the ^19^F capabilities of many modern NMR probe-heads.

## Electronic supplementary material

Below is the link to the electronic supplementary material.

(PDF 1055 kb)
