# Supplementary material for: A suite of 19F based relaxation dispersion experiments to assess biomolecular motions
Source: J Biomol NMR. 2020 Sep 30;74(12):753–66. doi: 10.1007/s10858-020-00348-4 (PMC7701166; doi:10.1007/s10858-020-00348-4)
Supplement: Supplementary file 1 — (PDF 1055 kb) [file 10858_2020_348_MOESM1_ESM.pdf]

Supporting information

Title:

A suite of ^{19}F based relaxation dispersion experiments to assess biomolecular motions.

Authors:

Jan H. Overbeck[1], Werner Kremer[1] & Remco Sprangers[1]

1 Department of Biophysics I, Regensburg Center for Biochemistry, University of Regensburg, 93053 Regensburg, Germany.

Correspondence can be sent to: Remco Sprangers, Department of Biophysics I, Regensburg Center for Biochemistry, University of Regensburg, 93053 Regensburg, Germany.

E-mail: remco.sprangers@ur.de

Journal of Biomolecular NMR

https://doi.org/10.1007/s10858-020-00348-4

Content **Page**

Content	Page
Supplementary Table 1: Parameters of chemical exchange for TmCsp	S3
Supplementary Table 2: Parameters of chemical exchange for the α7α7 half-proteasome	S4
Figure S1: ^{19}F CPMG experiment	S5
Figure S2: Monte Carlo simulation for CPMG experiments	S6
Figure S3: Monte Carlo simulation for on-resonance R1ρ experiments	S7
Figure S4: Monte Carlo simulation for off-resonance R1ρ experiments	S8
Figure S5: Monte Carlo simulation for a global fit of all RD experiments	S9
Figure S6: Global fit of CPMG, on-resonance R1ρ and off-resonance R1ρ experiments	S10
Figure S7: Comparison of on-resonance R1ρ data	S11
Figure S8: 19F CPMG experiment for BTFA-labeled 35C mutant of the half-proteasome	S12
CPMG pulse sequence	S13
R1rho on-resonance pulse sequence	S17
R1rho off-resonance pulse sequence	S20

Supplementary Table 1 Parameters of chemical exchange for TmCsp

	p_F [%]	k_{ex} [s^{-1}]	$\lvert\Delta\omega_{W7}\rvert$ [ppm]	$\lvert\Delta\omega_{W29}\rvert$ [ppm]	R_2^{W7} [s^{-1}]	R_2^{W29} [s^{-1}]	R_1^{W7} [s^{-1}]	R_1^{W29} [s^{-1}]
CPMG	95.2 ± 0.1	1966 ±485	2.46 ± 0.27	0.47 ± 0.05	10.7 ± 1.6	7.2 ± 0.6	-	-
$R_{1\rho}$ on-resonance	96.6 ± 0.4	2286± 268	2.62 ± 0.18	0.51 ± 0.02	11.2 ± 0.7	8.6 ± 0.2	-	-
$R_{1\rho}$ 100 Hz off-resonance	94.3±0.3	1818 ± 280	-2.33 ± 0.55*	-	1.4 ± 16.7	-	1.5 ± 0.2	-
$R_{1\rho}$ 200 Hz off-resonance	94.7 ± 0.3	1544 ± 219	-2.25 ± 0.30*	-	18.9 ± 11.3	-	1.9 ± 0.3	-
$R_{1\rho}$ 300 Hz off-resonance	94.8 ± 0.5	1795 ± 395	-2.31 ± 0.37*	-	12.1 ± 16.6	-	1.9 ± 0.5	-
$R_{1\rho}$ 400 Hz off-resonance	87.0±7.4	724 ± 461	-2.37 ± 0.53*	-	37.7 ± 8.8	-	0.8 ± 0.4	-
$R_{1\rho}$ off-resonance (all)	95.0 ± 0.2	1922 ± 137	-2.33 ± 0.18*	-	7.5 ± 4.6	-	1.8 ± 0.2	-
Global fit (CPMG + $R_{1\rho}$)	94.8 ± 0.1	1737 ± 54	-2.31 ± 0.14*	0.43 ± 0.01	11.5 ± 1.7 (CPMG)<br>10.9 ± 0.9 ($R_{1\rho}$)	7.4 ± 0.1 (CPMG)<br>8.7 ± 0.2 ($R_{1\rho}$)	1.9 ± 0.1	
T1 (Inversion Recovery)	-	-	-	-	-	-	1.53	1.22

* For these values the sign of the chemical shift difference has been determined.

Supplementary Table 2 Parameters of chemical exchange for the α7α7 half-proteasome

	**p_A [%]**	**k_{ex} [s^{-1}]**	**|Δω| [ppm]**	**R_2^{18C} [s^{-1}] (CPMG)**	**R_2^{18C} [s^{-1}] ($R_{1\rho}$)**
293 K	94.1 ± 1.4	8314 ± 1153	0.57 ± 0.08	234.0 ± 0.8	250.1 ± 0.6
303 K	96.7 ± 0.7	4102 ± 366		170.8 ± 0.5	174.8 ± 0.4
313 K	97.3 ± 0.4	1767 ± 281		130.3 ± 0.5	124.6 ± 0.6
323 K	97.6 ± 0.3	1503 ± 215		90.0 ± 0.4	90.5 ± 0.2

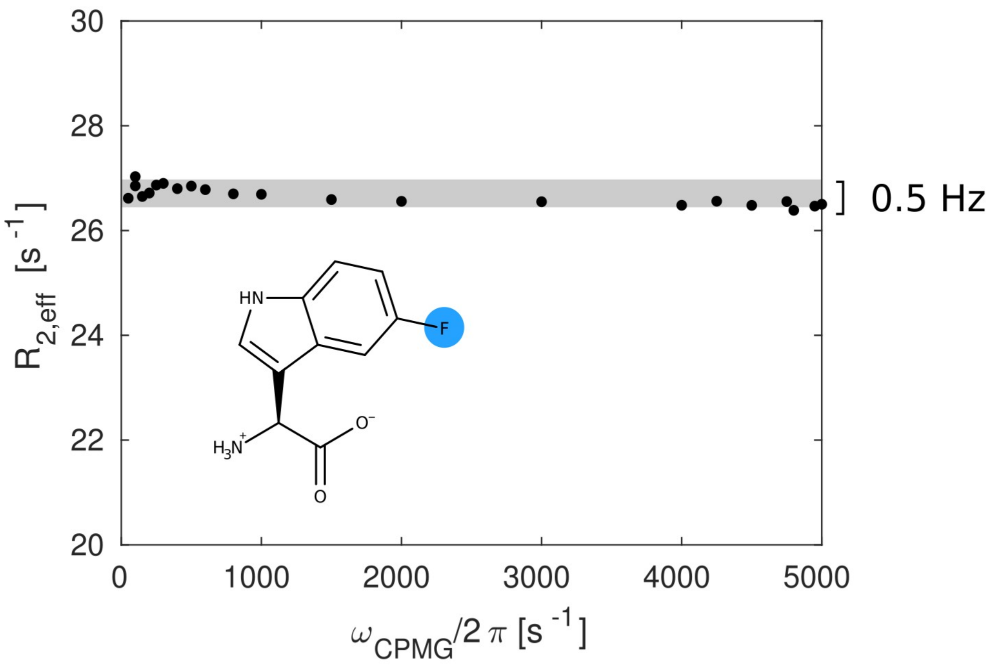

Figure S1: ^{19}F CPMG experiment CPMG experiment of 5-fluoroindole in glycerol at 298 K

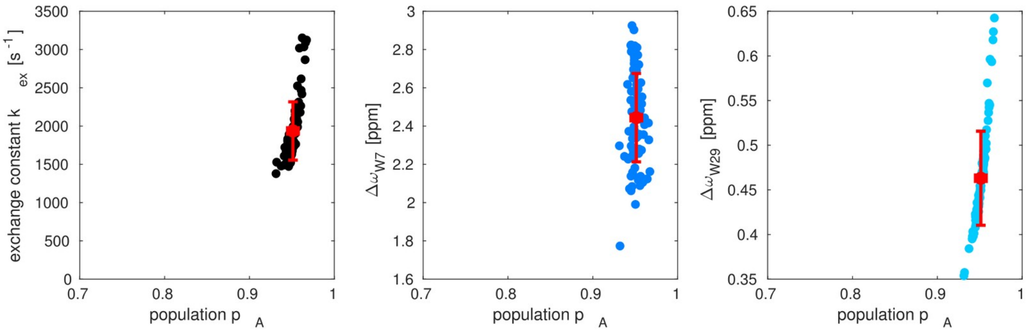

Figure S2: Monte Carlo simulation for CPMG experiments of 5FW-labeled TmCsp at 344 K at 500 MHz

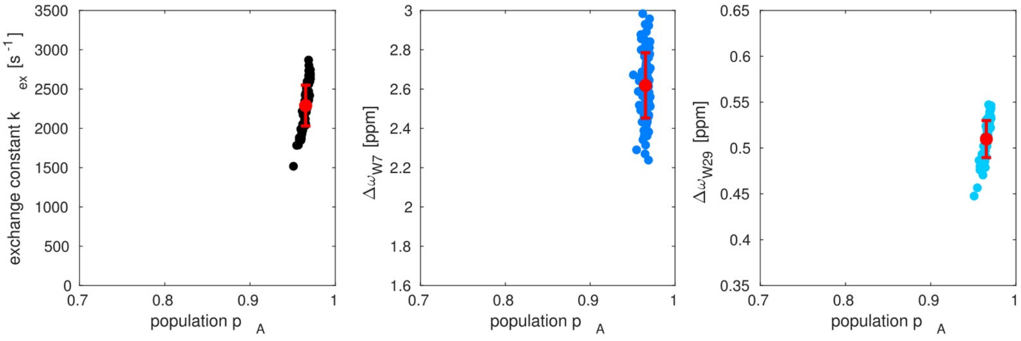

Figure S3: Monte Carlo simulation for on-resonance $R_{1\rho}$ experiments of 5FW-labeled TmCsp at 344 K at 500 MHz

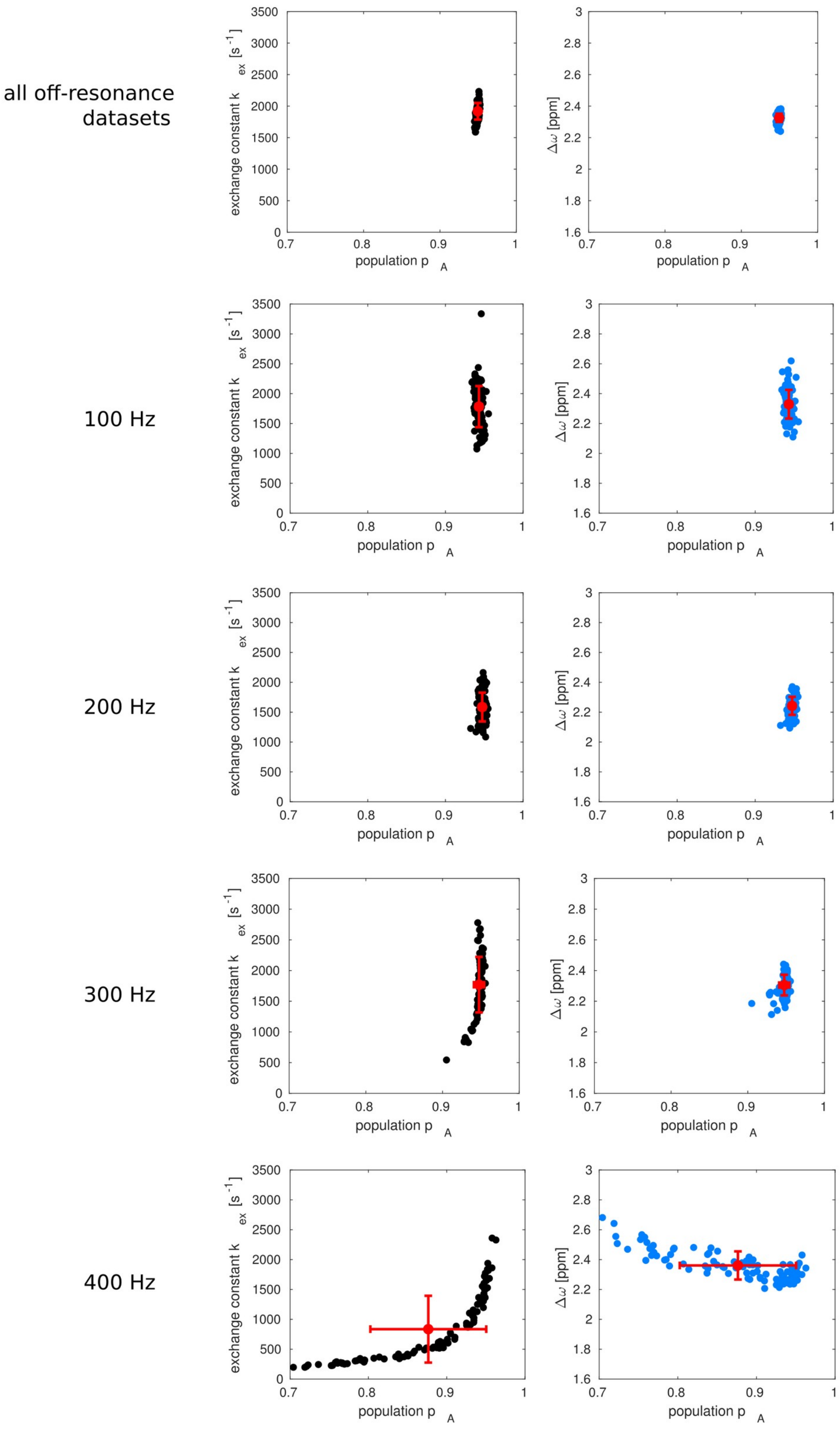

Figure S4: Monte Carlo simulation for off-resonance $R_{1\rho}$ experiments of 5FW-labeled TmCsp at 344 K and spin lock powers of 100 Hz, 200 Hz, 300 Hz and 400 Hz at 500 MHz.

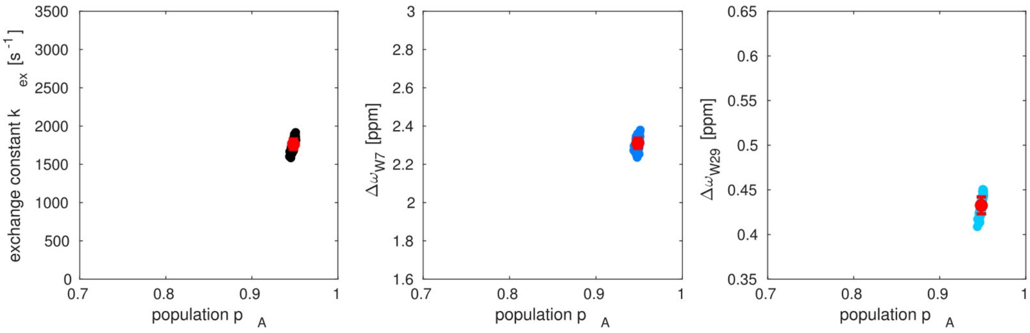

Figure S5: Monte Carlo simulation for global fit of CPMG, on-resonance $R_{1\rho}$ and off-resonance $R_{1\rho}$ experiments of 5FW-labeled TmCsp at 344 K.

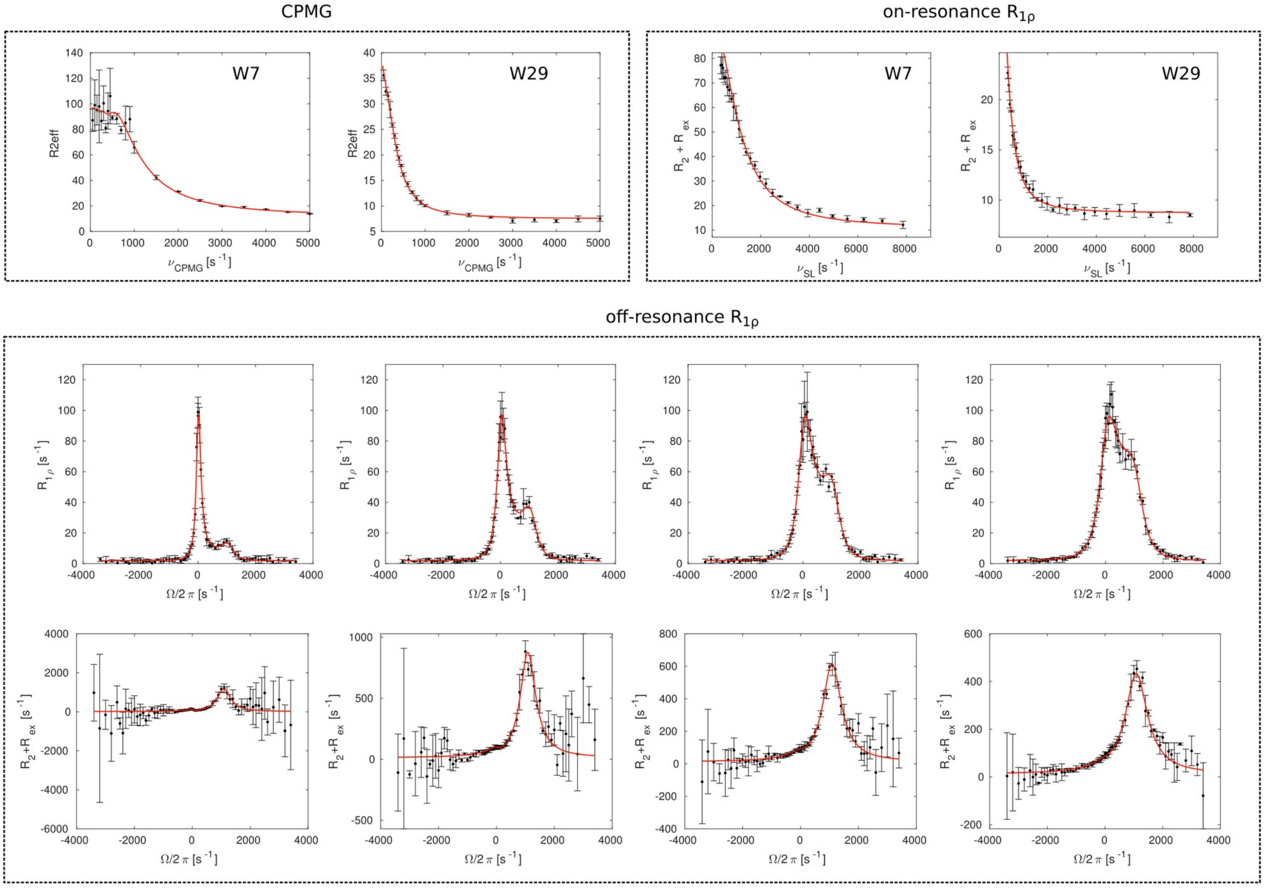

Figure S6: Global fit of CPMG, on-resonance $R_{1\rho}$ and off-resonance $R_{1\rho}$ experiments for TmCsp

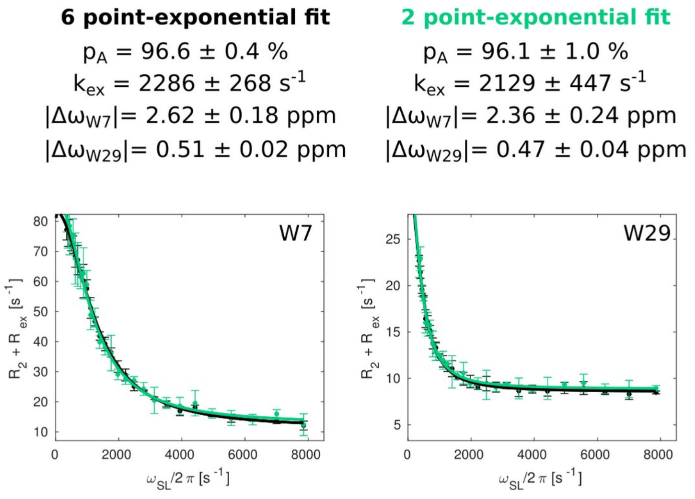

Figure S7: Comparison of on-resonance $R_{1\rho}$ data for TmCsp derived from exponential fits with 6 points (black) and 2 points (green). The spin lock times for 6 points were 0 ms, 4 ms, 8 ms, 16 ms, 32 ms and 48 ms. The spin lock times for 2 points were 0 ms and 16 ms (W7) and 0 ms and 32 ms (W29)

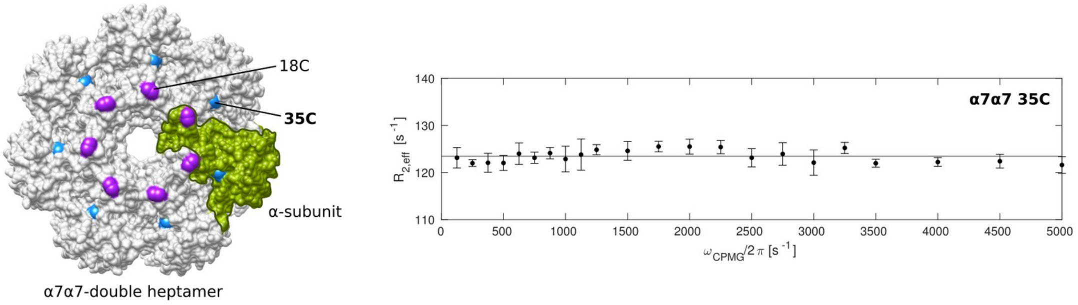

Figure S8: ^{19}F CPMG experiment for BTFA-labeled 35C mutant of the α7α7 half-proteasome

CPMG pulse sequence

```
/*
|
|  pulse sequence for 19F CPMG
|  including an anti-ringing sequence
|  Time_T2 (d15) is the length of the relaxation delay
|   tau_cpmg = Time_T2/4*ncyc is the time between two 180C pulses, where ncyc is the entry in the vclist
|  CPMG frequency = ncyc/Time_T2 (number of 360C 19F rotations per second)
|  CPMG frequency should not go over 5 kHz (or 2 kHz on BBO)
|  Jan Overbeck 2020
|
*/

/*--------------------------------
;     Parameters to set
; -------------------------------*/
;p1 = 90 degree fluorine hard pulse
;plw1 = watt power for hard fluorine

#include <Avance.incl>
#include <rs.incl>
#include <Grad.incl>

/*--------------------------------
;        define loop counter
; -------------------------------*/
define list<loopcounter> ncyc_cp = <$VCLIST>

"l2=0"                                                  ; loopcounter for CPMG experiments in vclist
"l5=0"                                              ; counter for number of pi pulses in actual CPMG
"l6=0"                  ; counter for number of pi pulses during compensation
"l7=0"                                                                      ; maximal number in vclist

"p2=p1*2"
"d11=30m"
"d13=4u"
"Time_T2=d15"

1 ze

; --- get largest entry in vclist ---
30m
"l7 = ncyc_cp.max"


/* --------------------------------
;     calculation of delay
; --------------------------------*/
2 30m
;"l5 = ncyc_cp[l2]"
```

```
"l5 = trunc(ncyc_cp[l2]+0.3)"

3 30m
if "l5 > 0"
{
"Tau_cpmg_a = (Time_T2/(4*l5)) - p1"
}
; --------------------------------

/*-------------------------------
; calculation number of
; heating compensation pulses
; for the next experiment
; -------------------------------*/
"l6 = l7 - l5"
; --------------------------------


/* --------------------------------
;     heating compensation
; --------------------------------*/


if "l6 > 0"
{
"Tau_cpmg_c = (Time_T2/(4*l6)) - p1"

4 Tau_cpmg_c
  (p1*2 ph2):f1
  Tau_cpmg_c
  lo to 4 times l6

5 Tau_cpmg_c
  (p1*2 ph2):f1
  Tau_cpmg_c
  lo to 5 times l6
}
else ; the recycle delay should stay constant
{
  Time_T2
}
100u

/* --------------------------------
;     add extra pi pulses
; to avoid starting magnetization
; --------------------------------*/
extral,100u
      (p1*2 ph2):f1
      100u
      lo to extral times 20
```

```
/* --------------------------------
;     this is the start
; -------------------------------*/

6 d1
50u UNBLKGRAD
p16:gp1
d16

p1 ph1



/* --------------------------------
;     CPMG block
; -------------------------------*/

;   d20
;   (p1*2 ph2):f1
;   d20

if "l5 > 0"
{
7  Tau_cpmg_a
   (p1*2 ph2):f1
   Tau_cpmg_a
lo to 7 times l5
}

if "l5 > 0"
{
8  Tau_cpmg_a
   (p1*2 ph2):f1
   Tau_cpmg_a
lo to 8 times l5
}

;   d20
;   (p1*2 ph2):f1
;   d20

/* --------------------------------
;     anti-ringing
; -------------------------------*/

p1 ph3


d13
p1 ph1
d13
```

```
p1 ph4
d13
p1 ph5

; -----------
  4u BLKGRAD
  go=2 ph31
  d11 do:f2 mc #0 to 2
  F1QF(calclc(l2,1))

HaltAcqu, 1m
exit

ph1=0 0 0 0 0 0 0 0 0 0 0 0 0 0 0 0 2 2 2 2 2 2 2 2 2 2 2 2 2 2 2 2 1 1 1 1 1 1 1 1 1 1 1 1 1 1 1 1
3 3 3 3 3 3 3 3 3 3 3 3 3 3 3 3
ph2=1 1 1 1 1 1 1 1 3 3 3 3 3 3 3 3 1 1 1 1 1 1 1 1 3 3 3 3 3 3 3 3 0 0 0 0 0 0 0 0 2 2 2 2 2 2 2 2
0 0 0 0 0 0 0 0 2 2 2 2 2 2 2 2
ph3=2 2 2 2 2 2 2 2 2 2 2 2 2 2 2 2 0 0 0 0 0 0 0 0 0 0 0 0 0 0 0 0 3 3 3 3 3 3 3 3 3 3 3 3 3 3 3 3
1 1 1 1 1 1 1 1 1 1 1 1 1 1 1 1
ph4=2 0 2 0 2 0 2 0 2 0 2 0 2 0 2 0 0 2 0 2 0 2 0 2 0 2 0 2 0 2 0 2 3 1 3 1 3 1 3 1 3 1 3 1 3 1 3 1
1 3 1 3 1 3 1 3 1 3 1 3 1 3 1 3
ph5=0 0 2 2 1 1 3 3 0 0 2 2 1 1 3 3 2 2 0 0 3 3 1 1 2 2 0 0 3 3 1 1 1 1 3 3 2 2 0 0 1 1 3 3 2 2 0 0
3 3 1 1 0 0 2 2 3 3 1 1 0 0 2 2
ph31=0 2 2 0 1 3 3 1 0 2 2 0 1 3 3 1 2 0 0 2 3 1 1 3 2 0 0 2 3 1 1 3 1 3 3 1 2 0 0 2 1 3 3 1 2 0 0 2
3 1 1 3 0 2 2 0 3 1 1 3 0 2 2 0


;pl1 : f1 channel - power level for pulse (default)
;p1 : f1 channel -  90 degree high power pulse
;p2 : f1 channel - 180 degree high power pulse
;d1 : relaxation delay; 1-5 * T1
;d11: delay for disk I/O                             [30 msec]
;d20: fixed echo time
;vc : variable loop counter, taken from vc-list
;ns: 8 * n or 64*n
;ds: > 128
;td1: number of experiments = number of values in vc-list
;define VCLIST
;this pulse program produces a ser-file (PARMOD = 2D)
;d20: decreases probe ringdown even in the absence of aring
```

R1rho on-resonance pulse sequence

```
/*
|
| On-resonance 19F R1rho as pseudo-3D
| with different SL lenghts read in via VPLIST
| and different SL powers read in via VALIST
|
| this pulse sequence can be used with hard pulses (default)
| or with adiabatic passages to the spin lock angle
|
| adiabatic_flg: use adiabatic passage to SL angle
|
| Pseudo-3D
| Jan Overbeck
| 2020
|
*/

/*--------------------------------
;     Parameters to set
; -------------------------------*/
;p11 : adiabatic pulse length, 4ms
;spnam4 : adiabatic ramp = tanhtan90
;sp4 : adiabatic ramp power, = pl25
;cnst28 : offset of SL in ppm
;p30 : maximum SL length
;p31 : heating compensation SL length
;p32 : spin lock lenght T_ex
;pl25 : spin lock power, = sp4
;VPLIST : list of spin lock lengths
;VALIST : list of spin lock powers  !in dB!

#include <Avance.incl>
#include <Grad.incl>

define list<pulse> plength = <$VPLIST>
define list<power> list1 = <$VALIST>

"p2=p1*2"
"d11=30m"
"l2=0"
"l3=0"

aqseq 312

1 ze
  "p30 = plength.max"

2 30m
/*--------------------------------
;    calculate SL delays
```

```
; -------------------------------*/
  "p32=plength[l2]"
  "p31=p30-p32"
; ---------------------------------

/* --------------------------------
;     heating compensation
; --------------------------------*/
if "p31 > 0.0"
  {
  1u fq=100(bf ppm):f1
  1u list1:f1
  (p31 ph1):f1
  }
; ---------------------------------

   d1
;50u UNBLKGRAD


/* --------------------------------
;  transfer to theta and SL
; --------------------------------*/
  30m
  1u fq=cnst28(bf ppm):f1

if "p32 == 0.0"
  {
#ifdef adiabatic_flg
    1u list1:f1
    (p11:sp4(currentpower) ph1):f1
#else
    1u pl1:f1
    p1 ph4
#endif
  }
else
  {
#ifdef adiabatic_flg
    1u list1:f1
    (p11:sp4(currentpower) ph1):f1
#else
    1u pl1:f1
    p1 ph4
#endif
  1u list1:f1
  (p32 ph1):f1
                                                                                    ; <-- this is the Spin Lock
}
;----------------------------------

/* --------------------------------
```

```
;  transfer back to z
; --------------------------------*/
#ifdef adiabatic_flg
    1u list1:f1
    (p11:sp5(currentpower) ph1):f1
#else
    1u pl1:f1
    p1 ph5
#endif
;------------------------------------

/* --------------------------------
;     anti-ringing
; --------------------------------*/
  1u pl1:f1
  p1 ph1
  d13
  p1 ph2
  d13
  p1 ph3
;------------------------------------

;  4u BLKGRAD
  go=2 ph31
  30m mc #0 to 2
  F1QF(calclc(l2,1))
  F2QF(calclist(list1,1))
;exit

HaltAcqu, 1m
exit

ph1=0
ph2=2 0
ph3=0 0 2 2 1 1 3 3
ph4=1
ph5=3
ph31=0 2 2 0 1 3 3 1
;ph31 = 0

;pl1 : f1 channel - power level for pulse (default)
;p1 : f1 channel -  90 degree high power pulse
;p2 : f1 channel - 180 degree high power pulse
;d1 : relaxation delay; 1-5 * T1
;d11: delay for disk I/O                             [30 msec]
;ns: 8 * n
;ds: 128
```

R1rho off-resonance pulse sequence

```
/*
|
| Off-resonance 19F R1rho as pseudo-3D
|
| with different SL lenghts read in via VPLIST
| and different SL offsets read in via FQ1LIST
|
| VPLIST: spin lock lengths
| FQ1LIST: offsets in Hz
|
| adiabatic_flg: use adiabatic passage to SL angle
|
| Pseudo-3D
| Jan Overbeck
| 2020
|
*/

/*--------------------------------
;     Parameters to set
; -------------------------------*/
;p11 = adiabatic pulse length, 4ms
;spnam4: adiabatic ramp = tanhtan90
;sp4: adiabatic ramp power, = pl25
;pl25: spin lock power, = sp4
;p32: spin lock lenght Tex
;VPLIST: list of spin lock lengths
;FQ1LIST: list of spin lock offsets !bf hz!

#include <Avance.incl>
#include <Grad.incl>

define list<pulse> plength = <$VPLIST>
define list<frequency> fqlist = <$FQ1LIST>

"p2=p1*2"
"d11=30m"
"l2=0"
"l3=0"

"cnst28=fqlist"
"p3 = p1*pow(10,(10*log10(plw1) - 10*log10(plw25))/20)"        ;90 degree SL pulse
"p6 = ((cnst28)/((1/(p3*4))))"                  ; spin lock offset / spin lock power
"p7 = atan(p6)"                              ; arc tan from this ratio = angle in rad
"p8 = p7*360/(2*PI)"                                                ; angle in degree
"p4 = p1*(1-p8/90)"                                               ; new pulse length

aqseq 312

1 ze
```

```
/*--------------------------------
; calculate hard pulse for offset
; dependent tip angle theta
; -------------------------------*/
2 30m
  "p30 = plength.max"
  "p32=plength[l2]"
  "p31=p30-p32"

  "cnst28=fqlist"

  "p6 = ((cnst28)/((1/(p3*4))))"                                ; spin lock offset / spin lock power
  "p7 = atan(p6)"                                           ; arc tan from this ratio = angle in rad
  "p8 = p7*360/(2*PI)"                                                          ; angle in degree
  "p4 = p1*(1-p8/90)"                                                          ; new pulse length
; --------------------------------

/* ---------------------------------
;     heating compensation
; --------------------------------*/
if "p31 > 0.0"
  {
  1u fq=100(bf ppm):f1
  1u pl25:f1
  (p31 ph1):f1
  ;print "heating compensation on"
  }
; ----------------------------------

   d1
;50u UNBLKGRAD

/* ---------------------------------
;  transfer to theta and SL
; --------------------------------*/

/*    cycle above&below plane     */
  1u fq=cnst29(bf ppm):f1
  1u pl1:f1
  p1 ph7
  p1 ph8


if "p32 == 0.0"
  {
  30m
#ifdef adiabatic_flg
    1u fq=fqlist:f1
    1u pl25:f1
    (p11:sp4(currentpower) ph1):f1
```

```
#else
    1u fq=cnst29(bf ppm):f1
    1u pl1:f1
    p4 ph4
#endif
  }
else
  {
  30m

#ifdef adiabatic_flg
    1u fq=fqlist:f1
    1u pl25:f1
    (p11:sp4(currentpower) ph6):f1
#else
    1u fq=cnst29(bf ppm):f1
    1u pl1:f1
    p4 ph4
#endif
  1u fq=fqlist:f1
  1u pl25:f1
  (p32 ph6):f1
                                                                             ; <-- this is the Spin Lock
}
; ----------------------------------

/* ---------------------------------
;  transfer back to z
; --------------------------------*/
#ifdef adiabatic_flg
    1u fq=fqlist:f1
    1u pl25:f1
    (p11:sp5(currentpower) ph6):f1
#else
    1u fq=cnst29(bf ppm):f1
    1u pl1:f1
    p4 ph5
#endif
; ----------------------------------

/*  cycle above&below plane BACK  */
  1u fq=cnst29(bf ppm):f1
  1u pl1:f1
  p1 ph7
  p1 ph8

/* ---------------------------------
;     anti-ringing
; --------------------------------*/
  1u pl1:f1
  1u fq=cnst29(bf ppm):f1
  p1 ph1
```

```
  d13
  p1 ph2
  d13
  p1 ph3
; ----------------------------------

;  4u BLKGRAD
  go=2 ph31
  30m mc #0 to 2
  F1QF(calclc(l2,1))
  F2QF(calclist(fqlist,1))
exit

HaltAcqu, 1m
exit

ph1=0
ph2=2 2 0 0
ph3=0 0 0 0 2 2 2 2 1 1 1 1 3 3 3 3
ph4=1 3
ph5=3 1
ph6=0 2
ph7=0
ph8=0 2
ph31=0 0 2 2 2 2 0 0 1 1 3 3 3 3 1 1

;pl1 : f1 channel - power level for pulse (default)
;p1 : f1 channel -  90 degree high power pulse
;p2 : f1 channel - 180 degree high power pulse
;d1 : relaxation delay; 1-5 * T1
;d11: delay for disk I/O                             [30 msec]
;ns: 16 * n
;ds: > 128
```
